# Efficient Detection of Repeating Sites to Accelerate Phylogenetic Likelihood Calculations

**DOI:** 10.1093/sysbio/syw075

**Published:** 2016-08-29

**Authors:** K. Kobert, A. Stamatakis, T. Flouri

**Affiliations:** 1 *Scientific Computing Group, Heidelberg Institute for Theoretical Studies, Schoβ-wolysbronnenweg 35, 69118 Heidelberg, Germany;*; 2 *Karlsruhe Institute of Technology, Institute for Theoretical Informatics, Postfach 6980, 76128 Karlsruhe, Germany;*

**Keywords:** Algorithms, maximum likelihood, phylogenetic likelihood function, phylogenetics

## Abstract

The phylogenetic likelihood function (PLF) is the major computational bottleneck in several applications of evolutionary biology such as phylogenetic inference, species delimitation, model selection, and divergence times estimation. Given the alignment, a tree and the evolutionary model parameters, the likelihood function computes the conditional likelihood vectors for every node of the tree. Vector entries for which all input data are identical result in redundant likelihood operations which, in turn, yield identical conditional values. Such operations can be omitted for improving run-time and, using appropriate data structures, reducing memory usage. We present a fast, novel method for identifying *and* omitting such redundant operations in phylogenetic likelihood calculations, and assess the performance improvement and memory savings attained by our method. Using empirical and simulated data sets, we show that a prototype implementation of our method yields up to 12-fold speedups and uses up to 78% less memory than one of the fastest and most highly tuned implementations of the PLF currently available. Our method is generic and can seamlessly be integrated into any phylogenetic likelihood implementation.

A typical approach in many phylogenetic analyses, such as Maximum Likelihood (ML) tree searches or Bayesian Inference (BI), is to evaluate the likelihood along each branch of a phylogeny with the assumption that mutation events follow a continuous time Markov process. Often, in such approaches, the repeated evaluation of the *phylogenetic likelihood function* (PLF) is by far the most costly operation. This is partially due to redundant calculations during the PLF evaluation that can be omitted. Accelerating the PLF is possible by taking into account that (sub-)trees with identical leaf labels (in our case nucleotides), identical branch lengths and the same model parameters always yield the same likelihood score or conditional likelihood values. Therefore, we can save computations by detecting repeating site patterns in the *multiple sequence alignment* (MSA) for a given (sub-)tree topology. From here on, we will refer to those repeating site patterns as *repeats*. Many phylogenetic inference tools such as PhyML (Guindon et al. 2010), RAxML (Stamatakis 2014), ExaBayes (Aberer et al. 2014), and MrBayes (Ronquist et al. 2012) utilize two methods exploiting this property to reduce computations. The first commonly used method consists in evaluating only the likelihood of unique columns of an MSA. Assuming only one set of model parameters for the entire MSA (i.e., unpartitioned analysis), identical sites yield the same likelihood. Therefore, the likelihood can be calculated by assigning a weight to each unique site, which corresponds to the site frequency in the original MSA. In the documentation of PHYLIP (Felsenstein 1993), Felsenstein refers to this method as *aliasing* (also frequently referred to as *site pattern compression*). The second standard technique for accelerating the PLF at *cherries*, that is, *inner* nodes whose descendants are both *tips* (or *leaves*), is to precompute the conditional likelihood entries for any combination of two states. Since there is a small, finite number of character states, those precomputed entries can be stored in a lookup table, and queried when needed, instead of repeatedly recomputing them. These two techniques are standard methods and are incorporated in virtually all PLF implementations providing faster computation times and often, considerable memory savings.

Apart from the aforementioned standard techniques, there are several studies on improving the run-time of the PLF. (Sumner and Charleston 2010) presented a method that relies on partial likelihood tensors. There, for each site of the alignment, the nucleotides at each tip node are iteratively included in the calculations. Let $s_i$ be the nucleotide for site $s$ at tip node $i$. The values are first calculated for $(s_1)$, then $(s_1,s_2)$, $(s_1,s_2,s_3)$, and so on, until $(s_1,s_2,s_3,\ldots,s_m)$ has been processed, where $m$ is the number of tip nodes. If the likelihood for another site $s'$ with $s_1'=s_1$, $s_2'=s_2$, and $s_3'\ne s_3$ is to be computed, the results for $s$, restricted to the first two tip nodes $(s_1,s_2)$, can be reused for this site. A lexicographical sorting of the sites is applied in an attempt to increase the number of operations that can be saved with this method. The authors report run-time improvements for data sets with up to $16$ taxa. For more than $16$ taxa, the performance of the method is reported to degrade significantly. Additionally, the authors measured the relative speedup of the PLF with respect to their own, unoptimized implementation and not the absolute speedup compared to the fastest implementation available at that time. (Izquierdo-Carrasco et al. 2011) mention the idea of using general site repeats for avoiding redundant PLF operations, but dismiss it as impractical because of the high book-keeping overhead. Instead, they only consider repeating subtree patterns consisting entirely of gaps, since they can be easily identified using bit-vectors, maintaining a low book-keeping overhead. In so-called “gappy” MSAs (alignments with a high percentage of gaps), the authors report a speedup of 25–40% and 65–68% memory savings on gappy alignments consisting of 81.53% and 83.4% gaps (missing data). The underlying data structure used for identifying such repeating subtree sites is called subtree equality vector (SEV) and was originally introduced by (Stamatakis et al. 2002). There, only homogeneous subtree columns are considered, that is, a repeat is only stored as such if all nucleotides in that subtree column are identical. This is again done to avoid the perceived complexity associated with finding general (heterogeneous) site repeats. By using this strategy, (Stamatakis et al. 2002) report a speedup of 19–22% for the PLF computation. Similar to (Sumner and Charleston 2010), (Pond and Muse 2004) devised a method for accelerating the likelihood computation of a site by storing and reusing the results obtained for a preceding site. Since only the results for one single site (the preceding site) are retained, an appropriate sorting of the sites is required. This column sorting approach is reported to yield speedups in settings where the PLF is evaluated multiple times for the same topology. The authors showed that sorting the sites to maximize the saving potential, can lead to run-time reductions from roughly 10% to over 80%, which corresponds to a more than 5-fold speedup. However, the authors also note that an ideal algorithm for PLF calculations would reuse all previously computed values from all sites and not just the neighboring ones. Furthermore, the optimal column sorting relies on solving the NP-hard traveling salesman problem and relies on the tree topology. Thus, to construct a polynomial-time algorithm, a search heuristic—that may yield sub-optimal results—is used. This means that the proposed column sorting may not yield the maximum amount of savings. (Larget and Simon 1998) propose another algorithm that considers site repeats. To identify repeated sites, at every node their method builds one bit-mask (that is in size linear to the number of taxa in the tree) for each site in the alignment. However, since this process relies on constructing and manipulating large bit-vectors at every node, and relies on sorting them for finding identical entries, it incurs a high computational overhead. (Valle et al. 2014) present another method that focuses on positive selection analysis, and that also deploys a variation of site repeats to accelerate the PLF. The authors implemented it into a redesigned, optimized version of CodeML [from the PAML package (Yang 2007)] called FastCodeML and tested its performance against the original CodeML package. Their method, which is specific to codon models and limited to fixed tree topologies, gives speedups of up to 5.8 over the sequential version of CodeML.

Here we show that it is possible to reduce memory requirements and attain a substantial acceleration of the PLF by generalizing the aliasing and cherry precomputation techniques. This can be achieved by detecting all conditional likelihood entries at *any* node in the tree, that yield identical likelihood values. Computing these entries only once is sufficient to calculate the overall tree likelihood or any of the omitted (duplicate) entries. The algorithm we present can be applied to both fixed and changing tree topologies. To have a practical application, such an algorithm must exhibit certain properties. First, the overhead incurred by finding repeats must be relatively small such that the overall PLF execution is faster. Second, the book-keeping overhead must be small such that it does not increase the PLF memory footprint. Third, the algorithm and the corresponding data structures must be flexible enough to allow for *partial* tree traversals. When evaluating new tree topologies via some tree rearrangement procedure (e.g., *nearest neighbor joining*, *subtree pruning and regrafting*), not all conditional likelihood vectors (CLVs) need to be updated. An efficient method for calculating repeats must take this into account and analogously only update the necessary data structures for the partial traversal (i.e., a subset of conditional likelihoods). Thus, the overall goal is to minimize the book-keeping cost for detecting repeats such that the memory usage and run-time is favorable. Furthermore, it is also necessary to consider hardware related issues such as nonlinear memory accesses which may lead to cache misses and expensive loads from RAM. For that reason, the absolute speedup of a new algorithm should be determined by using a highly optimized software for PLF calculations and not toy implementations.

We present a new, *simple* algorithm that satisfies the efficiency properties described above; it detects identical sites at *any* node of the phylogenetic tree and not only at the (selected) root, and thus minimizes the number of operations required for likelihood evaluation. It is based on our linear-time and linear-space (on the size of tree) algorithm for detecting repeating patterns in general, unordered, unrooted, $n$-ary trees (Flouri et al. 2013, 2014). To obtain the desired run-time improvements, we present an adapted version of this algorithm for the PLF that reduces book-keeping overhead and relies on two additional properties of phylogenies as opposed to general *multifurcating* (or $n$-ary) trees. First, we assume a *bifurcating* (binary) tree (although multifurcating trees can be used by arbitrarily resolving multifurcations). Second, the calculation of the so-called conditional likelihood depends on the transition probability of one state to another. These probabilities are not generally the same for different branches in the tree. Thus, we only consider identical nucleotide patterns to be repeats if they appear at the tips of the same (ordered) subtree. We test the performance of our method against PLL (Flouri et al. 2015)—a library derived from RAxML (Stamatakis 2014)—which offers one of the most highly optimized PLF implementations available. In particular, we show that a prototype implementation of the PLF, that uses our method, consistently outperforms the PLL/RAxML PLF by a factor of 2–12. In addition, the memory requirements are significantly lower, with cases where up to 78% less memory is required in comparison to RAxML. For the theoretical part of this article and the sake of simplicity, we assume that genetic sequences only contain the four DNA bases (i.e., A, C, G, T). However, the approach we present can be easily adapted to any number of states (e.g., degenerate DNA characters with gaps or protein sequence data). The data sets we use for benchmarking our method are empirical DNA data sets that *do* contain gaps and ambiguous characters.

## Algorithm

First, we introduce the notation which we will use throughout the article. A tree $T=(V,E)$ is a connected acyclic graph, where $V$ is the set of nodes and $E$ the set of *edges* (or *branches*), such that $E \subset V \times V$. We use the notation $(u,v) \in E$ to refer to an edge with end points $u,v \in V$, and $\theta_{u,v}$ to denote the parameters associated with it (such as branch length and evolutionary rate). The set Tip($T$) comprises the tip nodes. We use $T_u$ to denote a subtree of a (rooted) tree $T$ rooted at node $u$.

### The PLF

Before we introduce our method, it is necessary to give a brief description of PLF computations. The likelihood is a function of the states $\sigma$, the transition probabilities $P$ for all branches, and the equilibrium frequencies of the states $\mathbb{\pi} = \bigcup_{\forall s \in \sigma}\{ \pi_s \}$. In his seminal paper, (Felsenstein 1981) introduced the *pruning* algorithm, which is a dynamic programming approach for calculating the likelihood of a given tree $T$. The method iteratively computes all *conditional* (or *partial*) likelihoods $L^i_u(s)$, that is, the likelihood for a subtree rooted at node $u$, for site $i$, assuming the state at node $u$ is $s$, via a post-order (bottom-up) traversal of the tree. Such a traversal always performs the first computation at a cherry and conducts computations at a node only after both its children were visited. Now, let us assume an MSA of $n$ sites (columns) and $m$ sequences constructed from $s$ states (e.g., four for nucleotide data). The conditional likelihood $L^i_u(s)$ of any tip node $u \in {\rm Tip}(T)$ with the sequence $x=x_1x_2\ldots x_n$ is defined as

$$L_{u}^{i}(s)=\left\{ \begin{array}{cc} 1 & \;:\;s\in x_{i}\\ 0 & \;:\;s\notin x_{i} \end{array}\;\right.$$

where $x_i$ is the set of observed states for site $i$. In the case of *inner* nodes (i.e., $u \notin {\rm Tip}(T)$), the conditional likelihood for site $i$ is defined as

(1) $$\begin{array}{c} L_{u}^{i}(s)=(\sum_{\forall s_{v}\in \sigma }P_{s\mapsto s_{v}}(\theta_{u,v})L_{v}^{i}(s_{v}))(\sum_{\forall s_{w}\in \sigma }P_{s\mapsto s_{w}}(\theta_{u,w})L_{w}^{i}(s_{w})), \end{array}$$

where $P_{x\mapsto  y}(z)$ is the probability of generating a substitution from state $x$ to $y$ given parameter $z$, and $v$ and $w$ are the two descendants of $u$. We write the CLV entries for all possible states at a particular site $i$ of node $u$ as

(2) $$L^{u}(i)=\underset{\forall s\in \sigma }{⋃}L_{u}^{i}(s).$$

Finally, the overall likelihood $L$ for a rooted tree $T$ with root node $r$ is computed as

(3) $$L=\prod_{i=1}^{n}\sum_{\forall s\in \sigma }L_{r}^{i}(s)\pi_{s}.$$

We evaluate the overall likelihood of an unrooted binary tree at branch $(u,v)$ as

(4) $$L=\prod_{i=1}^{n}(\sum_{\forall s_{u}\in \sigma }L_{u}^{i}(s_{u})\pi_{s_{u}}\sum_{\forall s_{v}\in \sigma }P_{s_{u}\mapsto s_{v}}(\theta_{u,v})L_{v}^{i}(s_{v})).$$

For more details on the PLF, see (Felsenstein 2004).

### Site Repeats

Let $T_u$ be the subtree of $T$ that presents the evolutionary relationships among the taxa at tip nodes ${\rm Tip}(T_u)$. We denote the sequence of the $i$-th taxon $x^i = x^i_1 x^i_2 \ldots x^i_n$. Two sites $j$ and $k$ are called *repeats* of one another *iff*$x^i_j = x^i_k$ for all taxa $i$, $1 \leq i \leq |{\rm Tip}(T_u)|$, in $T_u$.

**Obervation** 1.*Let $u$ be a node whose two direct descendants (children) are nodes $v$ and $w$. Then, two sites $j$ and $k$ are repeats in $T_u$ if and only if $j$ and $k$ are also repeats in $T_v$ and $T_w$.*

**Figure 1.**
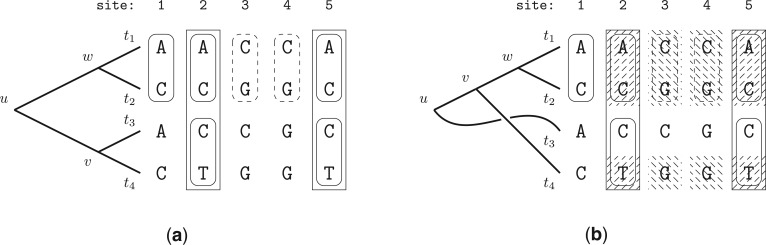
a) Sites 1,2, and 5 form repeats at node $w$ as they share the same pattern A C. Another repeating pattern is located at sites 3 and 4 (C G) for the same node. Note that node $v$ also induces a subtree with pattern A C at the tips. However, since branch lengths can be different than for the subtree rooted at node $w$, the conditional likelihoods may differ as well. Analogously, sites 2 and 5 are site repeats for node $v$ as they have the same pattern C T, and hence the conditional likelihood is the same for those two sites. Finally, sites 2 and 5 form repeats for node $u$ (A C C T). b) Repeats are not necessarily substrings of MSA sites. For this particular tree topology, node $v$ has two sets of repeats: sites 2 and 5 (A C T) and sites 3 and 4 (C G G). The repeats are not contiguous in the alignment columns.

Based on this observation, we can formulate the algorithm for detecting site repeats in binary phylogenetic trees. However, before we formalize the algorithm, let us consider Figure 1. From Observation 1, we see that the only repeating sites at the root node (node $u$), are sites $2$ and $5$. This is obviously correct, since both have the nucleotide pattern A C C T at the tips.

### Calculating Repeats

The method we propose identifies site repeats at each node via a bottom-up (post-order) traversal of the tree, meaning that a node is processed once the repeats for its two children have been determined. As tip nodes maintain only the trivial repeats of all sites that show a common character (for DNA, A, C, G, or T), the method always starts at a cherry. By construction, a cherry always exists in any binary tree, and assuming four nucleotide states, there are 16 possible combinations of homologous nucleotide pairs in the sequences of its two tip nodes. To assign a unique identifier to each nucleotide pair, we use a bijective mapping $\tau : \hat{\sigma} \times \hat{\sigma} \to \{1,2,\ldots,\hat{\sigma}^2\}$, where $\hat{\sigma}$ corresponds to the set of *observed* states (four for nucleotides or 16 when considering ambiguities and gaps). When dealing with DNA data, nucleotide states are typically encoded into integer values. Most (if not all) phylogenetic inference tools use the *one-hot* (also called *1 out of N*) encoding, which ensures that the binary representations of encoded nucleotides have exactly one bit set (e.g., A$\mapsto 1$, C$\mapsto 2$, G$\mapsto 4$, and T$\mapsto 8$). This is beneficial, because ambiguities, which are typically represented as disjoint unions of nucleotide characters (e.g., R$ =$A, G), can be encoded as the bit-wise OR (or sum) of the corresponding nucleotide codes (e.g., R$\mapsto 5$). Assuming that the sequence data is already encoded into integer values, we outline the method for identifying repeats at each of the three possible node types: cherry nodes (both its descendants are tip nodes), tip–inner nodes (one descendant is a tip and the other an inner node), and inner–inner nodes (both descendants are tip nodes).

#### Tip–tip (cherry) case.—

**Figure 2.**
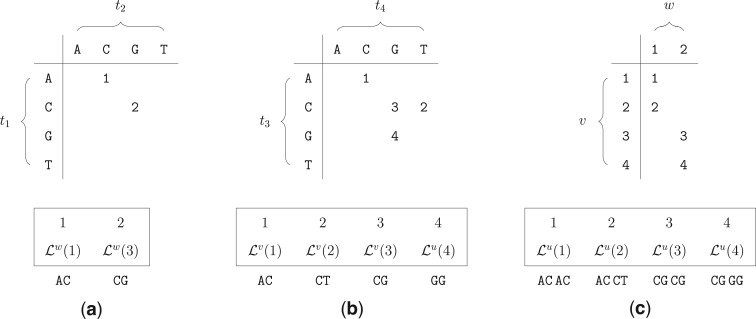
Identifier associations of nodes $w$ (a), $v$ (b), and $u$ (c) for the tree from Figure 1a. The respective lists at the bottom store the corresponding CLVs that are computed for each unique identifier. Table (a) shows that node $w$ requires two likelihood computations (sites 1 and 3), whereas the remaining sites are repeats of those two. Tables (b) and (c) show the corresponding information for nodes $v$ and $u$.

Let $x^v$ and $x^w$ be the (encoded) sequences of length $n$ at the two children $v$ and $w$ of the parent node $u$. Each site $i$ of $u$ is assigned the *identifier*$\phi_u(i) = \tau(x^v_i,x^w_i)$. By construction, this function assigns the same identifier to all sites $j$ which are repeats of site $i$ in $T_u$ [by Observation 1, $x^v_j=x^v_i$, $x^w_j=x^w_i$, and thus $\tau(x^v_j,x^w_j) = \tau(x^v_i,x^w_i) = \phi_u(i)$]. Figure 2 illustrates the assignment of identifiers to nucleotide pairs at the tips for the example in Figure 1. For every identifier $k \in \{ \phi_u(i)\ |\ 1 \leq i \leq n\}$, we compute the CLV entries of only the first site that was assigned $k$. These entries correspond to the unique CLV values for node $u$. By Observation 1, if a site $j$ is a repeat of site $i$ (i.e., both sites are assigned the same identifier), then the method can either (i) copy the CLV from site $i$ (run-time saving), or (ii) completely omit the likelihood value, since it can always retrieve it from site $i$ (run-time *and* memory saving). Furthermore, by Observation 1, we know that each repeat is identified by this method.

#### Tip–inner and inner–inner cases.—

We proceed analogously to detect repeats at nodes for which at least one child is an inner node. Again, let $u$ be the parent node and $v$ and $w$ the two child nodes for which we already computed all repeats. Further, let $\phi_v(i)$ and $\phi_w(i)$ be the respective identifiers of $v$ and $w$ at site $i$. In case one of the two nodes, say $v$, is a tip, then its identifier $\phi_v(i)$ is set to the encoded value of the observed states at site $i$. A site $j$ of node $u$ is a repeat of some other site $i$, *iff* the child node identifiers $\phi_v(i)$ and $\phi_w(i)$, are identical (one-to-one) with the identifiers $\phi_v(j)$ and $\phi_w(j)$. Therefore, the problem of detecting repeated sites is equivalent to finding duplicate records (in our case “pairs of identifiers”) in a hash table or index structure. Here, for simplicity, we use a multilevel table structure for indexing the identifiers. Let $\textit{vmax}$ be the maximum value of $\phi_v(i)$ and $\textit{wmax}$ the maximum value of $\phi_w(i)$ over all sites $i$. Those values represent the number of unique sites at nodes $v$ and $w$, and hence there cannot be more than $\textit{vmax}\times\textit{wmax}$ unique sites at $u$. Thus, we construct a $\textit{vmax}\times\textit{wmax}$ integer matrix $M$, where element $M_{\phi_v(i),\phi_w(i)}$ indicates that either (a) the identifiers $\phi_v(i)$ and $\phi_w(i)$ were not observed for another site before (in which case $M_{\phi_v(i),\phi_w(i)} = 0$), or (b) they were observed for a site which was assigned the identifier $M_{\phi_v(i),\phi_w(i)}$. Therefore, we query the element $M_{\phi_v(i),\phi_w(i)}$ for each site $i$ of $u$. If it is the first time a particular site is observed, we create a new, unique identifier $\textit{ident}$, and assign it to $M_{\phi_v(i),\phi_w(i)}$ and $\phi_u(i)$. In practice, *ident* is a simple counter initialized to one, which we increase each time a new pair of (so far unique) identifiers is detected. The CLV for site $i$ is then calculated and stored at position $\textit{ident}$ of an array called LH. Any subsequent site $j$ with identical child node identifiers as site $i$ [i.e., $\phi_v(j)=\phi_v(i)$, $\phi_w(j)=\phi_w(i)$], yields a nonzero value when querying entry $M_{\phi_v(j),\phi_w(j)}$. The retrieved value $M_{\phi_v(j),\phi_w(j)}$ is the identifier of site $i$ and we use it to (a) set the identifier of site $j$ [i.e., $\phi_u(j) \leftarrow M_{\phi_v(j),\phi_w(j)}$], and (b) retrieve its CLV entries from $\textit{LH}(M_{\phi_v(j),\phi_w(j)})$.

**Figure 3.**
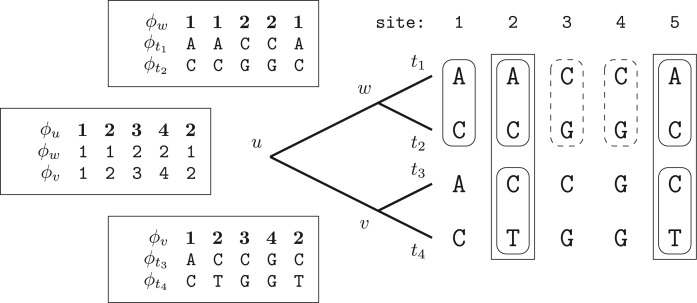
Identifiers (here $\phi_x$) are shown for every site of the alignment at every node in the tree. As we have already observed, sites $2$ and $5$ are repeats at node $u$, and thus, have been assigned the same identifier. For simplicity, identifiers at tip nodes are represented as nucleotide bases.

**Figure 4.**
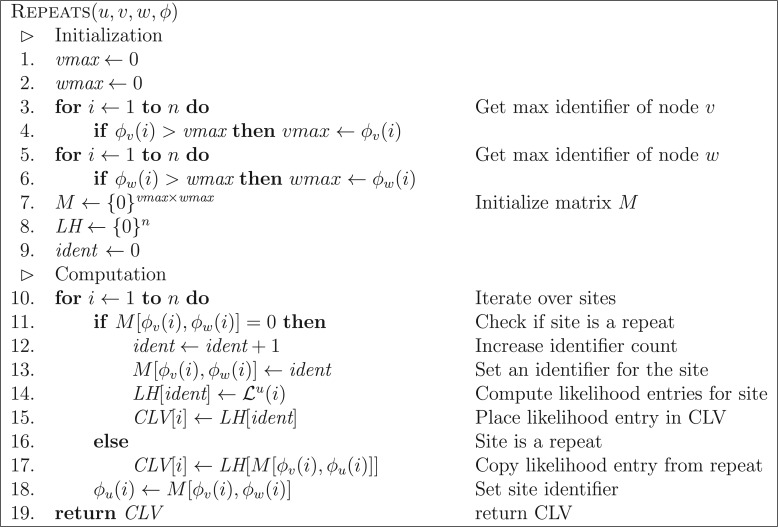
The algorithm to compute the CLV of a parent node $u$. The most costly operation is the calculation of the CLVs, expressed here as ${\cal L}^u(i)$ [see Equation (2)]. The algorithm minimizes the number of calls to this function by taking into account site repeats.

Figure 2c demonstrates the identifier calculation at node $u$ for the example tree and MSA from Figure 1, whereas Figure 3 shows the combined overall result. The listing in Figure 4 outlines the algorithm REPEATS$(u,v,w,\phi)$, which calculates the CLV for a node $u$ given child nodes $v$ and $w$, taking into account site repeat information. We will use the basic algorithm REPEATS to gradually build the complete method, that performs a post-order traversal over all nodes of tree $T$, and which incorporates the memory saving technique to reduce memory requirements. For that, we first explain how our method maintains the lookup table $M$ and the additional auxiliary data structures, and then we describe in detail the memory saving technique.

#### Lookup table.—

Since our focus is on an efficient implementation of the algorithm, we need to consider some technical issues in more detail. First, matrix $M$ (defined in algorithm REPEATS) can, in the worst case, become quadratic in size with respect to the number of sites in the alignment. This is unfortunate, since filling $M$ affects overall asymptotic run-time. However, in terms of practical space requirements, $M$ may be allocated only once and subsequently be reused for each inner node. A linear list *clean* with one entry per MSA site, can be used to keep track of which entries are *valid*, that is, contain identifiers assigned to sites of the current node, and which entries are *invalid* and contain identifiers assigned to the sites of a preceding node. After assigning an identifier $i$ to a site of node $u$, which we store in the array $M$, for example, at position $d$, we also store the pair $(d,u)$ in array *clean* at position $i$. When we process a different node, say $v$, and by chance, decide to assign the same identifier $i$ to some site, and again, by chance, the location for which we have to query matrix $M$ is $d$, the element $\textit{clean}[i]$ helps us distinguish between valid and invalid records in $M$. Invalid records are treated equivalently to empty records and are overwritten. Further, in the actual implementation we limit the size of $M$ to a constant maximum size. We implement this limit to adapt the impact of the quadratic complexity for filling $M$. Additionally, as $M$ grows larger (i.e., we move closer to the root of the tree), it becomes increasingly less likely to encounter repeats in the alignment. Thus, if the product of maximum identifiers for two child nodes at some node $u$ (i.e., $\textit{vmax} \times \textit{wmax}$) exceeds our threshold for the size of $M$, we do not calculate repeats any more. Instead, the CLV entries are calculated separately for all sites as in standard PLF implementations. In other words, if calculating repeats becomes disadvantageous, repeat calculations are omitted. This allows to trade repeat detection overhead, against PLF efficiency. In section “Computational Results,” Table 2 gives an overview of the size of $M$ for different data sets. Other methods with fast lookup times, such as hashing the pairs of identifiers, may work equally well as a constant size lookup table and, in fact, reduce the memory footprint and, consequently, cache misses. The constant size matrix $M$ defined here was chosen for simplicity of presentation and implementation.

#### Memory savings.—

**Figure 5.**
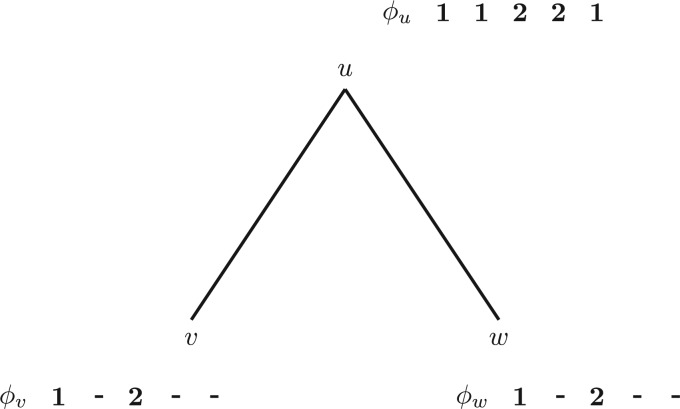
Not all sites are needed for the likelihood calculation at parent node $u$. According to the identifiers of this example, sites 2 and 5 are repeats of site 1, and site 4 is a repeat of site 3. Therefore, the CLVs at sites 2, 4, and 5 do not need to be computed nor stored, as the CLV for sites 2 and 5, and site 4, of node $u$ can be copied from sites 1 and 3.

Notice that, given algorithm REPEATS, not all entries in the CLVs of child nodes $v$ and $w$ are needed to calculate the CLV at the parent node $u$. In particular, the CLV entries at site $i$ for nodes $v$*and*$w$ are only needed if the CLV at site $i$ must be computed for $u$ (see Fig. 5). In fact, the CLV entries that are necessary to keep for any node (e.g., for computing the CLV of its parent node), are the ones stored in array $\textit{LH}$. This array is filled by executing algorithm REPEATS and contains only the unique CLV entries. Hence, we can replace the CLV array with the *LH* array, and, when needed, access the entries of site $i$ from $\textit{LH}[\phi_u(i)]$. In practice, this observation allows us to reduce the memory footprint of the PLF significantly, as each CLV entry stores more than one single or double precision floating point value. For example, RAxML stores one double precision floating point number (typically 8 bytes) per DNA character and per evolutionary rate for each CLV entry. Typically, the $\Gamma$ model of rate heterogeneity is used [see (Yang 1994)] with four discrete rate categories. Thus, the memory footprint of a standard PLF algorithm for a MSA with $n$ sequences of length $m$ is $8\times4\times4\times(n-2)\times m$ bytes. On the other hand, storing the site identifiers at each node only requires a single, unsigned integer per site. Thus, the memory required for storing CLVs without compression is $4\times 4=16$ times higher than that of the site identifier list.

**Figure 6.**
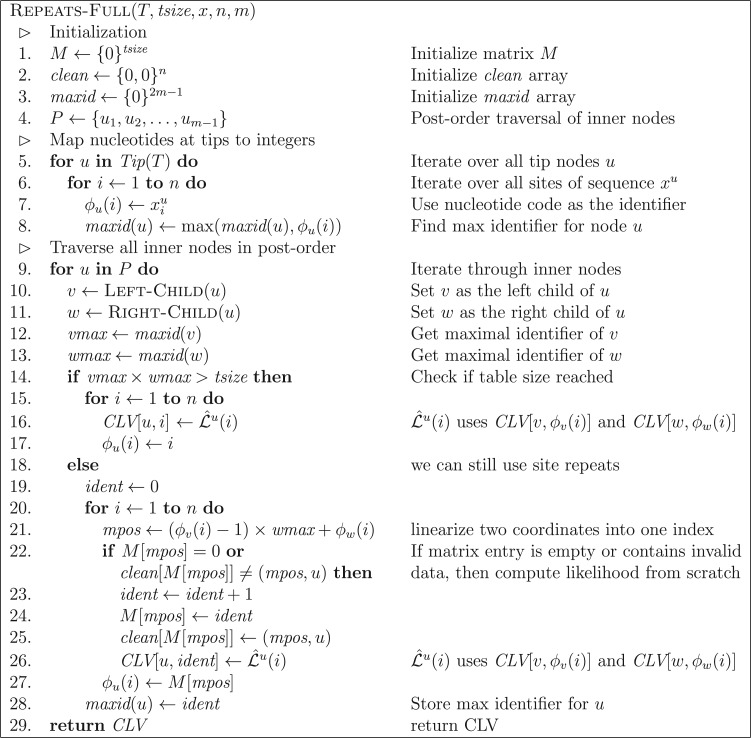
Full description for computing all CLVs of a tree $T$ with the memory saving technique and site repeat detection. Input parameters are a tree $T$ of $m$ taxa, the encoded sequences of size $n$ (denoted $x^u$ for the sequence at tip node $u$), and the size $\textit{tsize}$ of the matrix $M$ used for indexing site repeats. The algorithm computes only the necessary CLVs required for evaluating the likelihood of tree $T$ and skips PLF calls on repeated sites.

Thus, despite the fact that we need additional data structures, and hence space for keeping track of site identifiers, the memory requirements (if we do not store duplicate CLV entries) are smaller than those of standard production level tools (Flouri et al. 2015; Stamatakis 2014). While the identifiers are not the only additional data structures required for the actual implementation of the algorithm, the above argument indicates that storing fewer CLV entries can save substantial amounts of RAM. The overall algorithm, with memory savings and a bounded $M$, is given by algorithm REPEATS-FULL in Figure 6. One of the main differences compared to the snippet of Figure 4, is the introduction of a new array ($\textit{maxid}$) which stores the maximal identifier assigned to each of the $2m-1$ nodes of the rooted tree $T$ (assuming $T$ has $m$ tip nodes). Thereby, we eliminate the run time ${\cal O}(n)$ required for finding the maximal identifiers of the two child nodes (lines 3–6 in Fig. 4) at the cost of ${\Theta}(m)$ memory. The second difference is that we can no longer use the original set ${\cal L}^u(i)$ for the CLV entries of a site $i$ at a node $u$. This is due to the memory saving technique that omits the computation and storage of duplicate CLV entries as illustrated in Figure 5. The problem here is that the CLV entries of the two children $v$ and $w$ may not reside at position $i$ because repeats might have occurred. Instead, by construction of the identifiers $\phi_v(i)$ and $\phi_w(i)$, the correct values can be found at entries $L_v^{\phi_v(i)}$ and $L_w^{\phi_w(i)}$. To take this into account, we define a new CLV $\hat{{\cal L}}^u(i)$ analogously to Equation (1) as:

$$\begin{array}{cc} {\hat{L}}^{u}(i) & \;=\underset{\forall s\in \sigma }{⋃}(\sum_{\forall s_{v}\in \sigma }P_{s\mapsto s_{v}}(\theta_{u,v})L_{v}^{\phi_{v}(i)}(s_{v}))\\ & \;\;(\sum_{\forall s_{w}\in \sigma }P_{s\mapsto s_{w}}(\theta_{u,w})L_{w}^{\phi_{w}(i)}(s_{w})). \end{array}$$

Note that, in the algorithm REPEATS-FULL, $L_v^{\phi_v(i)}$ and $L_w^{\phi_w(i)}$ are extracted from $\textit{CLV}[v,\phi_v(i)]$ and $\textit{CLV}[w,\phi_w(i)]$.

**Observation 2 (Run-time).** *Algorithm* REPEATS-FULL *computes all site repeats, and the corresponding CLVs, in linear time with respect to the size of the alignment (number of sites $\times$ number of sequences), provided the allocated table $M$.*

This observation holds by inspection. For a description of the general, linear-time and *linear-space* algorithm that identifies all repeats in arbitrary $n$-ary trees and forests, see Section 3a of (Flouri et al. 2014). Since that algorithm relies on several sorting steps using bucket sort—a linear-time sorting method not based on the comparison model—it could, in practice, be slower than the method we present here, which only queries a lookup table. To compute all repeats, our method requires quadratic space (with respect to the number of sites) in the worst case, which, however, is allocated only once and reused for each node. When less memory is allocated, our method skips repeats identification for nodes where the product of unique site repeats for its two child nodes is larger than the entries of the allocated lookup table (i.e., low number of repeats). This is advantageous, since the overhead of repeat identification for such nodes could potentially cancel out the run-time savings.

Finally, when creating parallel implementations of the PLF that take advantage of our method, it is important to have in mind that sites do not necessarily have the same computational cost. A simple, shared-memory parallelization scheme that uniformly distributes the computation of a node’s CLV entries across threads requires a modification, as not all entries need to be computed, and therefore, distributing the computation uniformly, could end up in load imbalance. However, when identifying repeated entries, the memory saving technique can be adjusted to *weigh* the computational cost for each entry by assigning a weight of (i) one to entries that require calculation, and (ii) the proportion of time required to copy a CLV entry compared to calculating it for entries that will only be copied (repeats). Therefore, we can divide the load across threads such that the total weight is equally distributed. This can be trivially accomplished with an additional loop over the calculated weights. For more involved parallelization schemes, as presented by (Kobert et al. 2014), that are suitable for large scale computing, (Scholl et al. 2016) discuss a number of heuristic approaches for distributing load balance across computing nodes.

## Computational Results

We implemented a prototype of our algorithm in a new, low-level implementation of the PLL (Flouri et al. 2015) (which we refer to as LLPLL), that does not make use of the highly optimized PLF of PLL, but allows for a straightforward implementation of our algorithm. To demonstrate the applicability of our method under different settings, we created two implementations; one suitable for Site Repeats with Dynamic changing Topologies (SRDT) and one suitable for fixed, Site Repeats with Constant Topologies (SRCT). The first variant (SRDT) assumes no prior knowledge of the site repeats of a tree topology, and therefore, computes them before each PLF call. This variation is required for tree space exploration as site repeats change every time the tree topology is modified. The second variant (SRCT) computes site repeats only once as part of an initialization step. Assuming a constant, fixed topology, the PLF reuses the precomputed information from the initialization step at each invocation, as site repeats remain unchanged. This variant is suitable for applications where no tree exploration is performed, as, for example, in divergence time estimation (Heath et al. 2011) and model selection (Abascal et al. 2005), or during tree inference when parameters such as substitution rates or the $\alpha$ shape parameter of the gamma distribution are optimized.

To assess the performance of our method, we compared it with the sequential AVX-vectorized PLF implementation of the PLL which uses the same, highly optimized, PLF as RAxML. We selected PLL/RAxML because (i) it is our own code and hence we have a thorough understanding of it and (ii) it is currently among the fastest and most optimized PLF implementations available. This guarantees a fair comparison (i.e., determining the absolute speedup) and ensures that our method can truly be used in practice for speeding up state-of-the-art inference tools. We compare against two flavors of PLL: the plain version (we refer to it as PLL) and the memory saving SEV-based implementations of PLF (accessible through the $-U$ switch in RAxML) which we refer to as PLL-SEV. The latter is faster and requires less memory than the former in the case of particularly gappy alignments (Stamatakis et al. 2002). To obtain an accurate speedup estimate of our method, we vectorized the LLPLL likelihood function using AVX instructions. However, since LLPLL is in an early development phase, the PLL is still slightly faster by a factor of, approximately, 1.02–1.08 than LLPLL (without site repeats), as we show further. Despite this fact, we show that using our method, the LLPLL in its current state outperforms both PLL and PLL-SEV by up to a factor of 12.35. Our prototype implementation is available at http://www.exelixis-lab.org/web/software/site-repeats/.

### Data sets.—

For performing the experiments, we used one simulated (data set 7764) and nine empirical (corresponding to various bacterial, plant, and animal organisms) DNA data sets which are summarized in Table 1. All data sets contain gaps and ambiguous DNA characters and they have been used as benchmark data in previous PLF acceleration studies (Stamatakis and Alachiotis 2010; Stamatakis 2014). Table 1 also reports the percentages of gaps and site repeats in the alignments. The amount of gaps is important, as it affects the performance of the PLL-SEV implementation. The percentages of site repeats are given for an arbitrary rooting of the parsimony trees calculated for these data sets using RAxML. While the data set with 2000 taxa exhibits the lowest percentage of site repeats, it still has 86.95% repeats (which directly translate to identical conditional likelihood entries). We want to emphasize here that we did not choose these data sets based on their repeat percentages. In fact, the fraction of site repeats for each data set was previously unknown to us. All data sets used for testing, are available online at https://github.com/stamatak/test-Datasets.

**Table 1.** Summary of nucleotide data sets

Data set/sequences	59	128	354	404	500	994	1512	2000	3782	7764
Sites	6951	29,198	460	13,158	1398	5533	1577	1251	1371	851
Repeats(%)	92.04	91.78	94.65	96.49	89.43	94.63	90.09	86.95	94.18	87.62
Gaps(%)	44.24	32.48	14.71	78.92	2.25	71.39	3.02	12.65	2.70	20.60

*Notes:* For each data set, sites present the length of the provided MSA, and repeats denote the amount of sites over all nodes that are repeats of another site at the same node. The amount of repeats depends on the tree topology, the selected root, and the MSA. The (unrooted) trees were obtained by running a maximum parsimony tree search for each of the data sets and we randomly chose one node as the root to estimate the number of repeats.

### Experimental setup.—

For assessing the performance of our method, we conducted five types of experiments that cover the typical PLF use cases. First, we exhaustively assess the performance of full traversals for all possible rootings of the parsimony trees on two MSAs. Second, we assess the performance of full traversals on all 10 MSAs for a limited number of random rootings. Third, we evaluate the performance for partial traversals, that is, when not all CLVs need to be recomputed. Fourth, we assess the performance of our method on fixed tree topologies. In this setting, preprocessing of site repeats is done only once and not at each invocation of the PLF. Finally, using the three empirical multi-gene data sets in our collection, we determine the amount of memory required for maintaining the repeat tables when performing partitioned analysis. For the experiments, we used a 4-core Intel i7-2600 multi-core system with 16 GB of RAM. To eradicate the potential impact of server-side events such as context-switching or performance peaks of running processes, we always executed several (usually 10,000) independent likelihood computations. Also note that for all run-time comparisons, we focus purely on the PLF evaluation. Branch lengths and model parameters are fixed and remain unchanged as they do not impact the run-time of PLF.

Table 2 presents the memory savings due to site repeats together with the actual size of the lookup table for preprocessing all repeats. In the experiments, the size of the lookup table was bounded to 200 MB which corresponds to roughly 50 million entries (namely unsigned integer values). The actual memory for the lookup table was only allocated as needed. For most data sets, less than 200 MB of RAM was required. The notable exception is the data set containing 128 taxa, which requires 168.69 million entries (roughly 680 MB) to compute all site repeats. Since we bound the size of $M$ to 200 MB, not all repeats were preprocessed when analyzing this particular data set.

**Table 2.** Summary of memory requirements for each method to evaluate the PLF at a random rooting of the parsimony tree

Sequences	59	128	354	404	500	994	1512	2000	3782	7764
Sites	6951	29,198	460	13,158	1398	5533	1577	1251	1371	851
Memory PLL (MB)	53	474	24.5	680	93	707	312	328	678	875
Memory PLL-SEV (MB)	46	403	21.5	326	93	256	308	297	674	819
Memory SRCT/SRDT (MB)	32	303	7.5	202	34	164	104	120	171	298
Table size	5.3	168.69	0.07	23.5	0.82	6.6	2.9	2.4	2.8	0.87

*Notes:* The table size entry specifies the size of the lookup table $M$ of Algorithm Repeats-Full required to compute *all* possible repeats. It is presented in millions of entries (unsigned integers) and hence, its size in MB is four times as high as the presented numbers. The entries for “Memory SRCT/SRDT (MB) ” already include the table size in MB. For the data set with $128$ species only a table of 200 MB is allocated, as the table size is bounded by this number for our experiments.

## Exhaustive Evaluation of All Rootings

To get an initial estimate of the impact of distinct rootings on run-time, we used data sets 59 and 354 to evaluate the PLF at each *terminal* edge (an edge whose one end-point is a tip node) of their respective parsimony tree. This choice of rootings was selected because PLL requires that likelihood evaluations using full traversals of unrooted trees start at terminal edges. For each such rooting, we executed 10,000 independent PLF computations using each of the four implementations: the LLPLL (without site repeats), SRDT (LLPLL with our method), PLL, and PLL-SEV. For SRDT, we bounded the table size $M$ to 200 MB, which, according to Table 2, is sufficient to find all repeats for these two data sets.

Table 3 summarizes the results of the experiments. PLL is, on average, 1.08 times faster than LLPLL on data set 59 and 1.02 times faster on data set 354. PLL-SEV has a slightly better run-time than PLL, and is 1.17 times faster than LLPLL on data set 59 and 1.09 times faster on data set 354. The difference in speed between LLPLL and PLL/PLL-SEV can be explained by two factors. First, PLL is a highly optimized software for PLF calculations that was directly derived from RAxML, which in turn, has been developed and optimized for over 10 years, whereas LLPLL is in an early phase of development. Second, the standard optimization technique explained in the introduction, namely, the precomputation of conditional likelihoods for all combinations of two states with the subsequent querying from a lookup table (for tip–tip cases), is not implemented in LLPLL yet. This missing feature affects performance. Despite this fact, SRDT is on average 4.24 times faster than PLL and 3.92 times faster than PLL-SEV for the “gappy” data set 59. Similarly, for data set 354, SRDT is on average 5.87 times faster than PLL and 5.5 times faster than PLL-SEV. Note that, the standard deviation (SD) for SRDT is higher than PLL (and PLL-SEV), which is expected given that the amount of repeats changes with different rootings.

**Table 3.** Summary of run-times for evaluating the PLF at each terminal edge of the parsimony trees (data sets 59 and 354)

	Data set 59				Data set 354			
Implementation	Minimum	Maximum	Average	SD	Minimum	Maximum	Average	SD
PLL	$132.73$	$141.03$	$134.77$	$1.66$	$57.57$	$61.067$	$58.11$	$0.54$
PLL-SEV	$122.56$	$132.34$	$124.73$	$2.05$	$55.13$	$57.299$	$54.45$	$0.41$
LLPLL	$143.98$	$148.24$	$145.68$	$0.72$	$58.08$	$59.95$	$59.14$	$0.27$
SRDT	$27.04$	$37.25$	$31.8$	$2.53$	$8.81$	$12.22$	$9.9$	$0.64$

*Notes:* Presented are the minimum, maximum, and average run-times over all rootings for each of the four implementations, and for each data set, along with the SD of run-times among all rootings.

## Evaluation of a Sample of Rootings

For the comprehensive comparison of full tree traversal times between SRDT and PLL, we use nucleotide data sets ranging from 59 to 7764 taxa (see Table 1). We measured the run-times for 10 different rootings chosen at random, and which are not necessarily the same for SRDT and the PLL implementations. However, this comparison is sufficient given the SD among the run-times of different rootings computed for the exhaustive comparison of data sets 59 and 354. We again restricted the rootings to terminal branches, and for each of the 10 random rootings, we executed 10,000 full tree traversals. For each of the three implementations (SRDT, PLL, and PLL-SEV) we computed the average run-time over all 10 rootings. Table 4 shows the speedup of SRDT compared to PLL and PLL-SEV. As we see, SRDT is consistently at least three times as fast as PLL. In fact, the lowest observed average speedup is 3.3 compared to PLL and 2.08 compared to PLL-SEV. The maximal observed speedup was for the MSA with 354 taxa, where SRDT is 6.44 times faster than PLL. On the other hand, the largest decrease in speedups when comparing against PLL-SEV is observed for data sets 404 and 994 which comprise over 70% gaps. Note also that run-times for PLL-SEV were higher than for PLL on data sets with a low amount of gaps, such as 500, 1512, 3782, and 7764.

**Table 4.** Speedups obtained when evaluating the PLF using SRDT over PLL and PLL-SEV for each of the 10 data sets

Summary of speedups obtained using SRDT for a sample of rootings										
Data set	59	128	354	404	500	994	1512	2000	3782	7764
Speedup over PLL	4.25	3.75	6.44	6.04	3.39	5.12	3.67	3.06	4.76	3.3
Speedup over PLL-SEV	3.88	3.41	6.22	3.3	3.56	2.08	3.84	2.93	5	3.32

*Notes:* SRDT is consistently faster than both methods. Speedups are computed as fractions of average run-times over 10 random rootings.

## Partial Traversal Performance

**Figure 7.**
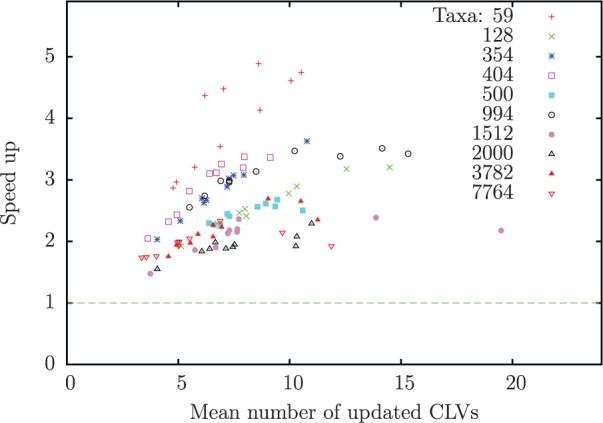
Run-time improvements of the SRDT method over LLPLL, plotted against the average number of CLV updates. Each data set is represented by 10 measurements for 10 different nodes.

In phylogenetic inference, it is not always necessary to perform full tree traversals to calculate the overall likelihood of a tree. In particular, when conducting BI or ML tree searches that deploy local topological updates using, for instance, nearest neighbor interchange or subtree pruning and regrafting moves, only the CLVs of a subset of tree nodes need to be recomputed. Depending on the topological update, those CLVs may be located at the inner part of the tree where the number of repeats is lower. We assessed the performance of our approach for this scenario by emulating partial CLV updates as follows. For each parsimony tree, we choose two adjacent nodes (sharing an edge) at random and place them in an empty list of nodes whose CLVs are to be updated. Next, with probability $P$, we choose whether to continue adding nodes to the list or stop (with probability $1-P$) the procedure. If we choose to add nodes, we randomly pick one (unvisited) adjacent inner node to each of the (at most) two nodes selected in the previous step, and add them to the list. The procedure is repeated until we choose to stop or until no single inner node is available anymore for selection. This pattern of CLV updates emulates the topological moves described by (Lakner et al. 2008) for BI. As mentioned before, in addition to the time spent in the PLF, other factors, such as branch length and model parameter optimization for ML, also contribute to the overall execution time. Here, we concentrate only on measuring the time for calculating the PLF. We used the method described above to simulate 10 partial CLV updates for each data set from Table 1. Each simulation consists of a path of nodes generated from a randomly chosen pair of adjacent nodes with probability $P$ set to 0.95. We calculated each CLV along the path 10,000 times and measured the total execution time for LLPLL and SRDT. Figure 7 presents the individual speedups for each data set and each simulation, plotted against the number of nodes updated for the particular simulation. We did not compare SRDT to PLL/PLL-SEV since a fair comparison requires that exactly the same partial updates are evaluated, which is difficult to achieve given the different internal structures of the two implementations. Therefore, the speedup for the partial updates is not the absolute speedup for different PLF implementations. Instead, our results show the relative speedup that can be achieved by incorporating our method into any PLF implementation.

## Performance on Fixed Topologies

Many phylogenetic tools use a fixed tree topology on which the likelihood is repeatedly evaluated while other parameters, such as substitution rates and branch lengths, are modified. Divergence times estimation (Heath et al. 2011) and model selection (Abascal et al. 2005) are typical representations for this setting. With fixed topologies, repeats can be precomputed once in an initialization step and then reused for subsequent PLF invocations. We tested our method under this setting, by evaluating the likelihood 10,000 times, rooted at 10 randomly selected terminal edges of the parsimony trees of each of the 10 data sets. We averaged the run-time over the 10 rootings and report the speedups of SRCT over PLL and PLL-SEV in Table 5. We computed the speedup for each data set as the ratio of the average run-time of PLL or PLL-SEV from section “Evaluation of a sample of rootings” divided by the average run-time of SRCT. Data set 994 yields the lowest speedup over PLL-SEV, which is due to the high number of gaps in the data set, followed by data set 2000 which has the lowest number of repeats. On the other hand, the highest speedup of SRCT over PLL-SEV was for data set 354 which has a high number of repeats combined with a low amount of gaps. As expected, the highest speedup of SRCT over PLL is observed for data set 404, since it has the highest amount of repeats among all data sets.

**Table 5.** Speedups obtained using the SRCT method which considers a fixed topology over the PLL and PLL-SEV

Summary of speedups obtained using SRCT when considering fixed topologies										
Sequences	59	128	354	404	500	994	1512	2000	3782	7764
Sites	6951	29198	460	13158	1398	5533	1577	1251	1371	851
Speedup over PLL	7.56	5.66	11.6	12.35	5.39	10	5.84	4.59	8.45	4.94
Speedup over PLL-SEV	6.89	5.16	11.2	6.74	5.66	4.03	6.12	4.39	8.85	4.96

## Partitioned Analyses

We performed partitioned analysis on the three empirical multi-gene data sets (59, 128 and 404) in our collection to compare the accumulated memory requirement of per-partition repeat tables against single repeat tables from unpartitioned analyses. This experiment is of high practical relevance because typical phylogenetic analyses are partitioned nowadays. Table 6 provides a summary of the per-gene partitions of each data set, the required per-partition table size for computing all repeats, and the per-partition speedups over PLL-SEV. For measuring the speedups, we used the same parsimony trees from the previous experiments, and computed an average run-time over 10 random terminal edge rootings. As in the rest of the experiments, the rootings may not be the same between PLL-SEV and LLPLL. Similarly to Table 2, per-partition table sizes are presented in millions of entries (unsigned integers). One important result is that the accumulated table size for each partitioned data set is considerably smaller than the table size required for processing single-partitioned data sets (see Table 2). Compared to their single-partition counterparts, dataset 59, 128 and 404, require 10, 13, and 6 times less memory for storing the per-partition tables. Not surprisingly, this is due to the way we index elements in the table (see line 21 of Figure 6). This kind of indexing may cause the table to grow quadratically to the maximum number of unique repeats at a node, which in turn, increases with the size of the alignment.

**Table 6.** Summary of per-partition table sizes for data sets 59, 128, and 404

Partition	Sites	Speedup	Table size	Partition	Sites	Speedup	Table size
Data set 59: Poaceae							
5.8S	160	6.64	0.001	ndhf1st	2183	4.82	0.24
cprs	364	2.78	0.053	phyb3rd	1182	4.18	0.08
gbss13rd	774	4.64	0.016	rbcl1st	1344	6.27	0.033
its2	264	2.25	0.06	rpoc23rd	680	3.39	0.038
Data set 128: Mammalia							
12S_rRNA	860	3.79	0.28	Cytb	1149	3.25	0.74
16S_rRNA	1275	3.89	0.36	EDG1	978	5.51	0.085
ADORA3	330	4.72	0.025	IRBP	1233	4.32	0.45
ADRB2	833	5.32	0.08	ND1	963	3.41	0.56
APOB	1335	4.14	0.47	ND2	1050	2.92	0.85
APP	717	4.12	0.09	ND3	349	2.76	0.1
ATP6	681	2.94	0.24	ND4L	297	2.77	0.057
ATP7A	684	4.26	0.095	ND4	1378	3.07	0.99
ATP8	213	2.43	0.048	ND5	1836	2.85	2.69
BDNF	582	5.59	0.024	ND6	555	2.9	0.23
BMI	340	5.30	0.0086	PLCB4	373	3.37	0.067
BRCA1	2934	3.85	2.38	PNOC	333	4.21	0.022
CNR1	993	6.06	0.069	RAG1	975	4.93	0.088
COX1	1557	3.82	0.77	RAG2	444	5.01	0.024
COX2	708	3.21	0.22	tRNAs	1341	3.84	0.46
COX3	804	3.35	0.24	TYR	426	4.37	0.028
CREM	468	4.28	0.04	ZFX	204	5.55	0.0022
Data set404: Poaceae							
ndhF	2161	4.2	1.17	rbcL	1437	5.06	0.28
rpoC2	802	2.94	0.24	phyB	1218	3.8	0.28
tRNA	1646	2.8	1.15	psbA	1020	3.56	0.002
rps4	588	5.56	0.0027	rps16	1025	2.84	0.032
adh1	852	3.37	0.03	matK	1581	4.43	0.22
its1, 5.8S, its2	828	2.63	0.33	—	—	—	

*Notes:* All table sizes are presented in millions of entries (unsigned integers) and indicate the size of the lookup table $M$ required to compute all possible repeats. When performing partitioned analysis, data set 59 requires a total of 0.521 million entries, data set 128 requires 12.88, and data set 404 requires 3.74. We also present the speedups over PLL-SEV for evaluating the PLF for each partition.

## Conclusion

The PLF is among the computationally most time-consuming functions in evolutionary biology and often constitutes the largest portion of total run-time in analyses involving PLF calculations. Especially in the era of genomics, where datasets comprise thousands or even millions of alignment sites (Jarvis et al. 2014; Misof et al. 2014), accelerating the PLF can save weeks or even months of CPU time.

We introduce a novel method for quickly identifying all repeating site patterns, and consequently, minimizing the number of operations required for evaluating the PLF. It is based on our linear-time and linear-space algorithm for identifying repeating subtrees in general labeled, unordered, $n$-ary trees. Our new method is optimized to work with phylogenetic trees (rooted or unrooted binary trees) and discards many of the hidden constants behind the complexity of the original method for $n$-ary trees. Moreover, its simplicity allows it to be incorporated into virtually any PLF implementation.

To measure the speed of the PLF during tree searches or model parameter optimizations, we compared a prototype implementation of our method against PLL—a library derived from the RAxML code—which uses one of the fastest and most highly tuned implementations of the PLF. Using empirical and simulated data, we measured the speedup under different, realistic settings. For fixed and dynamically changing tree topologies, we observe an up to 12-fold speedup. For partial CLV updates, that is, when only a small number of CLVs is recomputed due to a topological rearrangement of the tree, we still observe an up to 5-fold speedup. Furthermore, our method, *including* the book-keeping information for site repeats requires significantly less memory than PLL, sometimes up to 78% less. Using empirical data, we also show that the book-keeping storage requirements for partitioned analyses are significantly smaller than for unpartitioned analyses (up to 13 times less), which is particularly important for optimizing large phylogenomic analyses. Moreover, the table used for book-keeping information is flexible, and can be dynamically adjusted, for example, in an “auto-tuning” step, to determine the table size—and consequently the amount of identified repeats—that minimizes PLF run-time. When the allocated table size is exceeded (e.g., at a node with a small amount of repeated sites), our method will omit repeat identification for all subsequent nodes, as the amount of repeats decreases towards the root of the tree. This allows our method to omit repeat computations at nodes for which calculating them is disadvantageous.

## Supplementary Material

Data available from the Dryad Digital Repository: http://dx.doi.org/10.5061/dryad.8f0c8.
